# Theoretical studies on RNA recognition by Musashi 1 RNA-binding protein

**DOI:** 10.1038/s41598-022-16252-w

**Published:** 2022-07-15

**Authors:** Nitchakan Darai, Panupong Mahalapbutr, Peter Wolschann, Vannajan Sanghiran Lee, Michael T. Wolfinger, Thanyada Rungrotmongkol

**Affiliations:** 1 Program in Bioinformatics and Computational Biology, Graduate School, Chulalongkorn University, Bangkok, 10330 Thailand; 2 Department of Biochemistry, Faculty of Medicine, Khon Kaen University, Khon Kaen, 40002 Thailand; 3 Department of Theoretical Chemistry, University of Vienna, Währinger Strasse 17, 1090 Vienna, Austria; 4 Department of Chemistry, Faculty of Science, University of Malaya, 50603 Kuala Lumpur, Malaysia; 5 Research Group Bioinformatics and Computational Biology, Faculty of Computer Science, University of Vienna, Währinger Strasse 29, 1090 Vienna, Austria; 6 Center of Excellence in Biocatalyst and Sustainable Biotechnology, Faculty of Science, Chulalongkorn University, Bangkok, 10330 Thailand

**Keywords:** Molecular modelling, RNA

## Abstract

The Musashi (MSI) family of RNA-binding proteins, comprising the two homologs Musashi-1 (MSI1) and Musashi-2 (MSI2), typically regulates translation and is involved in cell proliferation and tumorigenesis. MSI proteins contain two ribonucleoprotein-like RNA-binding domains, RBD1 and RBD2, that bind single-stranded RNA motifs with a central UAG trinucleotide with high affinity and specificity. The finding that MSI also promotes the replication of Zika virus, a neurotropic Flavivirus, has triggered further investigations of the biochemical principles behind MSI–RNA interactions. However, a detailed molecular understanding of the specificity of MSI RBD1/2 interaction with RNA is still missing. Here, we performed computational studies of MSI1–RNA association complexes, investigating different RNA pentamer motifs using molecular dynamics simulations with binding free energy calculations based on the solvated interaction energy method. Simulations with Alphafold2 suggest that predicted MSI protein structures are highly similar to experimentally determined structures. The binding free energies show that two out of four RNA pentamers exhibit a considerably higher binding affinity to MSI1 RBD1 and RBD2, respectively. The obtained structural information on MSI1 RBD1 and RBD2 will be useful for a detailed functional and mechanistic understanding of this type of RNA–protein interactions.

## Introduction

RNA-binding proteins (RBPs) are key regulators of numerous cellular processes, mediating different aspects of co- and posttranscriptional gene expression. They contain well-defined RNA-binding domains (RBDs) that confer sequence- and/or structure-specificity for endogenous target RNAs^1^. Examples of evolutionary conserved RBDs are the RNA recognition motif (RRM), the heterogeneous ribonucleoprotein (hnRNP) K-homology (KH) domain, and the C3H1 zinc-finger (ZF) domain. These bind to a relatively restricted set of the primary RNA sequence space, often utilizing additional contextual traits such as RNA secondary structure or base compositional context for additional specificity^2^.

The Musashi (MSI) protein family comprises a group of RBPs that act as translational regulators and are involved in the maintenance and self-renewal of neuronal progenitor and stem cells^3^. They have been initially identified in the central nervous system, where they are involved in the regulation of Notch signaling by binding to the mRNA of its antagonist Numb^4^. While MSI proteins are typically expressed in stem cells^5^, they are absent in differentiated tissue. Being evolutionarily conserved among invertebrates^6,7^ and vertebrates^8^, there has been emerging evidence that MSI proteins mediate biological processes that regulate the initiation and progression of various cancer cells, including colorectal, breast, lung, and pancreatic cancers, as well as leukemias and glioblastoma^9^.

The MSI gene has been duplicated in vertebrates, resulting in the two paralogs Musashi-1 (MSI1) and Musashi-2 (MSI2), each containing two ribonucleoprotein (RNP)-type RNA recognition motifs (RRMs) in their N-terminal regions, followed by a poly(A)-binding protein region. While the structures of the mouse MSI1 and MSI2 RRMs have been solved^10–12^ the sequence identity of the regions containing the two RRMs in mouse MSI1 and MSI2 is remarkably high at 86%^13^, suggesting a common RNA target motif. For MSI1, this has been determined as (G/A)U_n_AGU (n = 1–3) by an in vitro selection approach (SELEX)^4^. NMR titration experiments with a series of RNA oligomers revealed that MSI1 RBD1 and RBD2 bind to GUAG and UAG motifs with high affinity^13,14^. These data are in line with cross-linking and immunoprecipitation (iCLIP) studies, which revealed the trinucleotide sequence UAG in a single-stranded structural context, predominantly in the 3'UTRs of mRNAs, as a core Musashi binding element (MBE)^15,16^. Likewise, quantitative fluorescence anisotropy assays confirmed the binding specificity of the UAG trinucleotide, while nucleotides outside this core MBE have limited contribution to the overall binding free energy^17^.

A different aspect of MSI pathobiology has been recently elucidated, i.e., the role of MSI proteins as host factors in viral infections, specifically their capacity to promote Zika virus (ZIKV) replication^18^. ZIKV is a mosquito-borne Flavivirus (MBFV) that has been circulating for decades in Africa and Asia, often being misdiagnosed as dengue. During a ZIKV outbreak in the Americas in 2015–2017, an unexpectedly high number of congenital malformations coupled with intrauterine growth restrictions, placental damage, and microcephaly has been associated with ZIKV infections^19^. While MBFVs are typically horizontally transmitted between arthropod vectors and vertebrate hosts, the capacity for transplacental passage aligns ZIKV with a handful of other MBFVs, including West Nile virus (WNV) and Powassan virus (POWV), that have been shown to cause placental infection and fetal neuropathology^20^. The presence of UAG-containing MBEs in the 3'UTRs of Flavivirus genomes, together with in vivo data revealing that MSI not only interacts with ZIKV RNA but also enhances viral replication, has led to the understanding that MSI is involved in ZIKV-induced neurotropism^18^. It has been hypothesized that MSI might stabilize viral RNA, thereby maintaining a sufficient RNA level that is not translated but subjected to purposeful exoribonuclease degradation^21^. The latter results in the production of short flavivirus RNA (sfRNA), which modulates cellular mRNA decay^22^ and antiviral interferon response^23,24^. While these findings highlight the instrumental role of MSI in virus-associated cytopathicity, the biochemical foundations and mechanisms of the MSI-mediated congenital neuropathology remain elusive.

Computational prediction of the structural accessibility of RNA binding motifs is a promising approach for the characterization of RNA-protein binding sites. This idea has been applied to several eukaryotic RBPs, resulting in the observation that target site accessibility almost always increases the ability to predict sequence-specific RBP–RNA binding^25^. We have recently addressed the question as to whether other Flaviviruses have a similar MSI-mediated neurotropic potential to ZIKV by analyzing the affinity of Musashi binding elements (MBEs) in 3'UTR regions to appear in a single-stranded structural context, which is a requirement for efficient MSI–RNA interaction^21^. To this end, we have shown that the structural accessibility of MBEs along viral RNA molecules can be used as a proxy for predicting MSI–RNA interactions, thereby assessing the neurotropic potential of viruses. By employing a thermodynamic model of RNA folding based on the ViennaRNA package^26^, we computed the average opening energy that is necessary to keep specific MBEs in an unpaired structural context, rendering them accessible for MSI RRM–RNA interaction. Our data highlighted that MBEs in the 3' untranslated region (3'UTR) of ZIKV are highly accessible for MSI binding, thereby corroborating earlier studies that addressed the neurotropic potential of flaviviruses and alphaviruses^20^.

**Figure 1:**
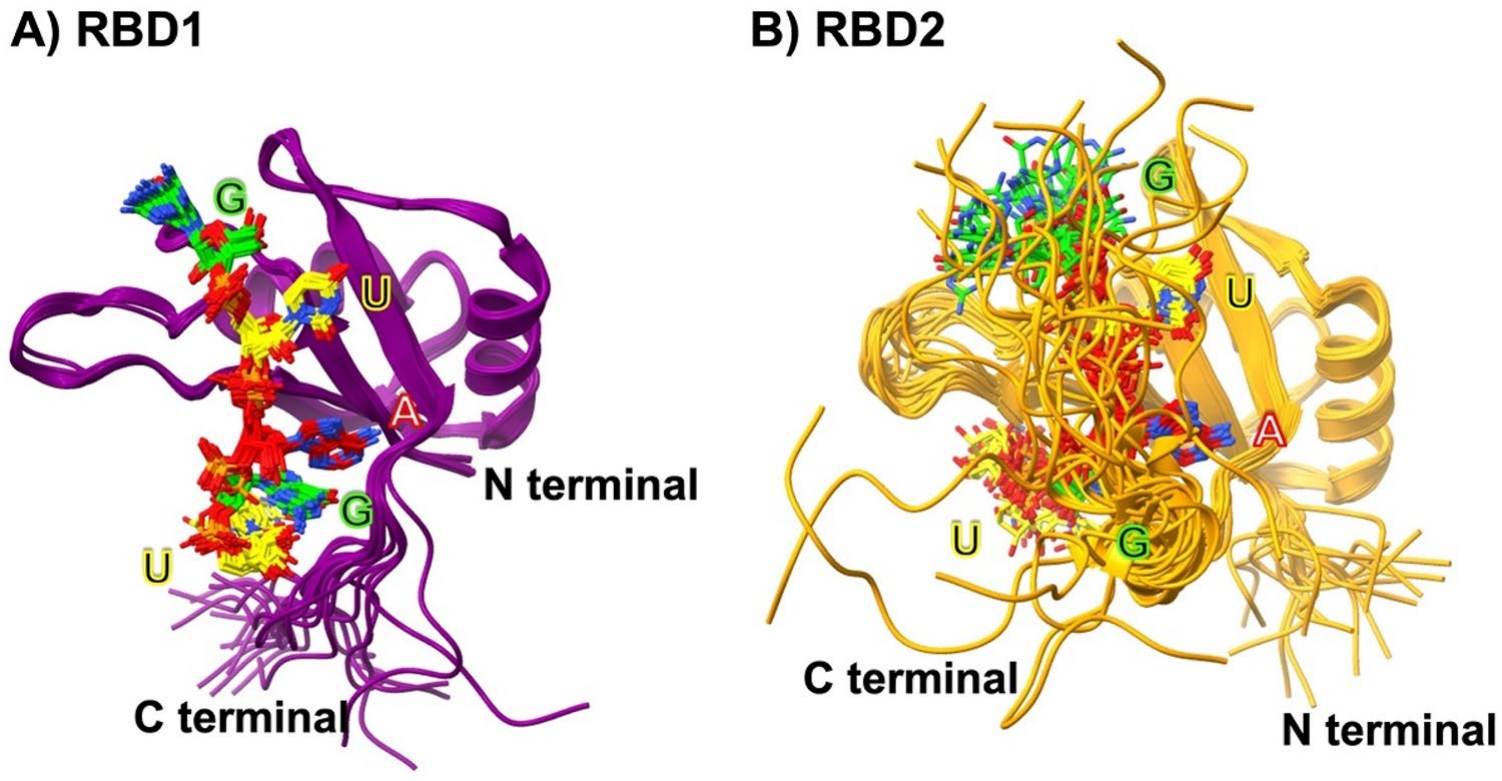
(**A**) Superimposition of the 20 NMR structures of MSI1 RBD1 (PDB ID: 2RS2) and (**B**) MSI1 RBD2 (PDB ID: 5X3Z) with the RNA pentamer GUAGU bound.

Here we follow up on this idea and model the 3D structure of MSI RBDs with Alphafold2. Subsequently, we investigate MSI–RNA association complexes, employing molecular dynamics (MD) approaches to gain more insight into the molecular traits of this type of RNP binding. Specifically, we focus on the published MSI1 RBD1–RNA complex and MSI1 RBD2–RNA complex (PDB IDs 2RS2^13^ and 5X3Z^14^), as shown in Fig. 1A and B, which were derived from NMR spectroscopy. Superimposition of MSI1 RBD1 and MSI1 RBD2 NMR structures and their sequence alignment is shown in Supplementary, Figures S1A and S1B. The RMSD between RBD1 and RBD2 structure is 0.997 Å. The RNA component of the complex comprises a canonical MBE with the pentamer sequence GUAGU. We set out to mutate individual RNA nucleotides to study the energetics of MSI1 binding to alternative RNA motifs. To this end, we selected three additional pentamers, i.e., GUUGU, GGAGU, and GAUGU, whose central trinucleotides exhibited high, medium, and low affinities, respectively, within the thermodynamic ensemble of ZIKV 3'UTRs^21^.

## Materials and methods

### Protein structure prediction with Alphafold2

AlphaFold2^27^ is an artificial intelligence (AI) approach for highly accurate protein structure prediction. In combination with MMseqs2^28^, a program for protein sequence search within large databases and generation of high quality protein sequence alignments, Alphafold2 is capable of simulating high accuracy structures for a wide range of proteins, for which structural data are unavailable. Here, we performed predictions for MSI1 RBD1 and MSI1 RBD2 using ColabFold^29^, which couples MMseqs2 and AlphaFold2 in publicly available notebooks that can be executed on the Google Cloud infrastructure. We were specifically interested in determining the protein structures in the apo form and comparing these to structures available through PDB. The sequences of MSI1 RBD1 and MSI1 RBD2 were retrieved from PDB IDs 2RS2 and 5X3Z. The first candidate structure (model 1) of both RBDs from ColabFold was selected as the initial conformation to assess GUAGU binding to both RBDs by MD simulations.

### Molecular dynamics simulations

The top five NMR structures of MSI1 RBD1/2:GUAGU complexes were retrieved from PDB IDs 2RS2 and 5X3Z. The LEaP module of AMBER16^30^ was used to construct complexes with three alternative RNA pentamers (GUUGU, GGAGU, and GAUGU) by modifying the central trinucleotides. The protonation states of RNA–protein complexes were computed using the PDB2PQR server at pH 7.4. The AMBER ff14SB and chiOL3 (OL3) force fields^30^ were employed for protein and RNA, respectively. According to standard procedures, the missing hydrogen atoms of each system were added by the LeaP module. The added hydrogen atoms were then minimized for 1000 steps by steepest descents (SD) and subsequently by 3000 steps of conjugated gradient (CG). Subsequently, solvation of each system was performed by TIP3P water molecules^31^ of approximately 6800 atoms for RBD1 and 7300 atoms for RBD2 in a periodic box at a distance of 12 Å apart from the protein surface, resulting in a box dimension of 63 × 70 × 62 Å^3^, and 70 × 66 × 63 Å^3^, respectively. The systems were neutralized using Na + counter ions. Periodic boundary condition with isothermal-isobaric ensemble (NPT) ensemble and a step-size of 2 fs for the simulation time were applied. The water molecules and ions were then minimized with 1000 steps of the steepest descent (SD) and continued with 3000 steps of the conjugate gradient (CG) method. The entire system was fully minimized in the last step by the same minimization process. All bonds with hydrogen atoms were constrained using the SHAKE algorithm^32^. MD simulations under periodic boundary conditions were performed five times for all systems using the AMBER16 software package^30^.

The MD simulation started by heating the system from 10 to 310 K. Next, the system was equilibrated at a constant temperature of 310 K. 100 ns MD simulation was performed under NPT conditions at 1 atm and 310 K. The last 20 ns MD trajectories were taken for structural and energetics analyses. Root-mean-square displacement (RMSD) and distance between the centers of mass of protein and RNA were calculated by the cpptraj module of AmberTools16^33^. The interactions between protein and RNA were visualized and analyzed using Discovery Studio Visualizer^34^. Additionally, the solvated interaction energy (SIE)^35^ method was applied to estimate the binding affinities of MSI1 RBD1/2 RNA complexes, and to determine the binding contribution of each nucleotide. SIE is an end-point physics-based scoring function that approximates the binding free energy from the force–field non-bonded interaction terms, continuum solvation, and configurational entropy linear compensation^35^. For each individual simulation, the SIE binding free energy of the complex was calculated over 200 snapshots from the last 20 ns (1000 snapshots in total) using the equation:

$${\Delta}{G}_{\rm bind} \, = \, {\upalpha} \, \times \, \left[{\text{E}}_{\rm c} \left({\text{D}}_{\text{in}}\right) \, + \, {\Delta{\text{G}}}^{\text{R}} \, + { \, \Delta{\text{E}}}_{\rm vdW} \, + \, {\upgamma} \cdot \, \Delta{\text{MSA}}\left(\rho\right)\right] \, + \, {\text{C}}$$

The binding affinity prediction was estimated by summation of Coulomb interactions (∆*E*_c_) and van der Waals interactions (∆*E*_vdW_), the electrostatic solvation contribution (∆*G*^R^), reaction field energy, and nonpolar desolvation energy. ∆*E*_c_ and van der Waals interactions of the bound state were calculated with AMBER ff14SB and OL3 molecular mechanics force fields. The electrostatic solvation contribution was carried out using the continuum dielectric model with a solute interior dielectric constant and a solvent dielectric constant. The reaction field energies were considered by the Poisson equation with the boundary element method program. The nonpolar desolvation was estimated by a linear proportionality with the change in the solute molecular surface area^35^. Note that the global proportionality coefficient associated with the loss of conformational entropy upon binding (α) is 0.104758, while the solute interior dielectric constant (D_in_) is 2.25. The molecular surface area coefficient (γ) is 0.012894 kcal/mol^−1^ Å^−2^, ΔMSA(ρ) is the difference in molecular surface area between the bound and free state of the protein and constant (C) is − 2.89 kcal/mol^−1^. These parameters were optimized by fitting to the absolute binding free energy^36,37^ The binding affinity values of the canonical RNA motif (GUAGU) and three modified RNA motifs (GUUGU, GGAGU, and GAUGU) with MSI1 RBD1 and MSI1 RBD2 from the SIE method were taken from the 200 snapshots of the last 20 ns of the five models of each system (1000 snapshots in total). For the amino acids involved in each nucleotide binding of the four RNAs, Δ*G*_bind,res_ calculations based on the MM/PBSA method were performed on the same series of 1000 snapshots.

## Results

### Structure prediction of MSI1 RBD1/2:GUAGU

**Figure 2:**
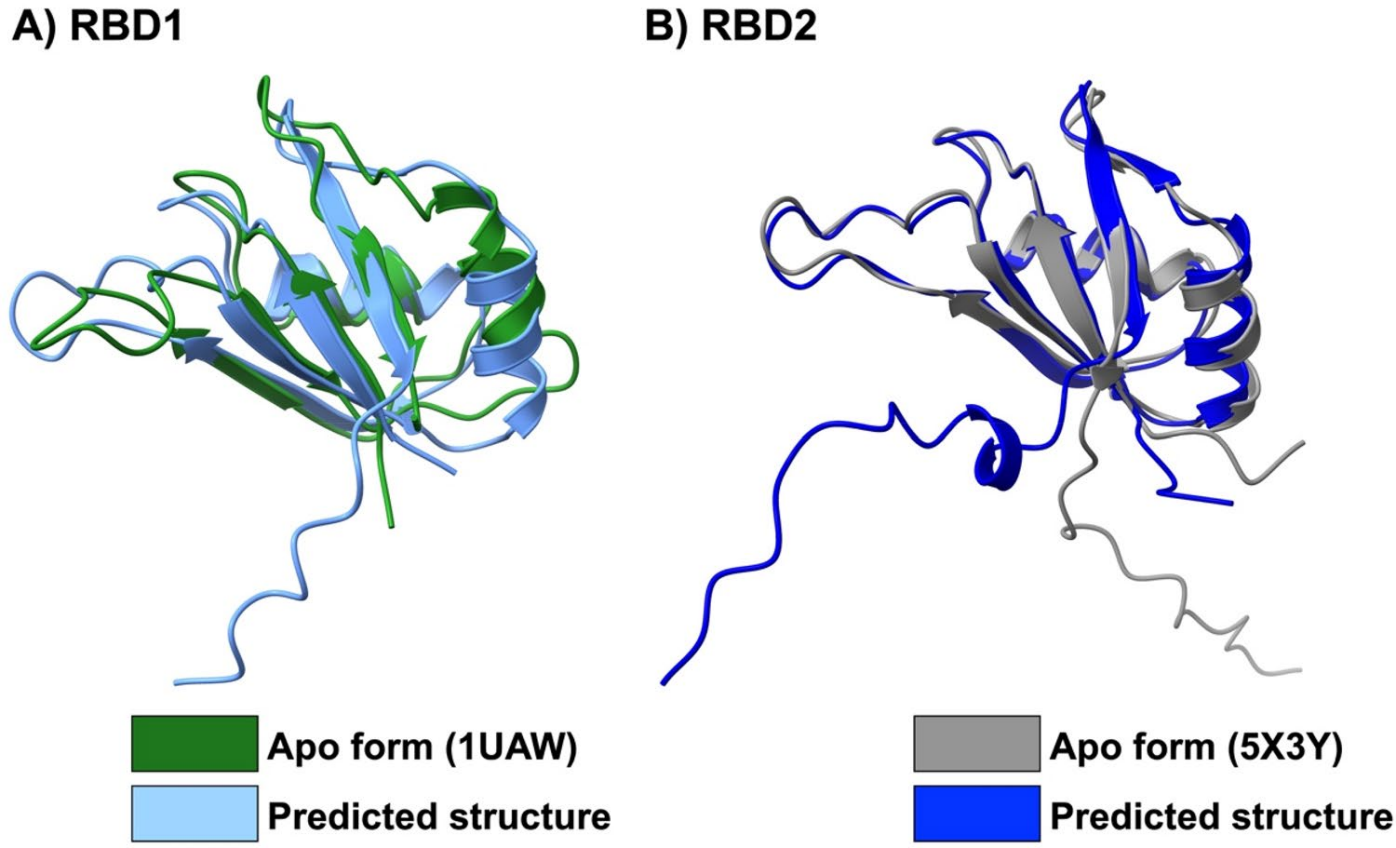
Superimposition of (**A**) MSI1–RBD1 and (**B**) MSI1–RBD2 from NMR structures in apo form and the predicted structure from Alphafold2.

For the five predicted structures of MSI1 RBD1/2 from Alphafold2, the number of sequences per position and the per-residue confidence metric (pLDDT) are used to determine the validity of the Alphafold2 results (Figure S2). For MSI1 RBD1, the core structure is covered by approximately 600 sequences at each position, while there are only approximately 100 sequences in the C terminal region (Figure S2A). Likewise, the model confidence at each position increases up to 90% and drops to 70% and 40%, respectively, at the flexible loops and C-terminal. Interestingly, all predicted structures of the five models of MSI1 RBD1 exhibit a similar structure, except in the C terminal region. A similar situation is found for the MSI1 RBD2 models, except that the model confidence at the two terminals is lower to some extent (Figure S2B). The core structure of the predicted models MSI1 RBD1/2 is comparable to the experimentally solved structures (PDB IDs 1UAW and 5X3Y), in particular, RBD2 with the RMSD value 1.777 and 0.837 Å, respectively (Fig. 2).

For further investigations, MD simulations of the protein–RNA association complex were performed. To this end, 100 ns MD simulations were applied on the complex between model 1 of the Alphafold2 simulations, and the canonical RNA pentamer (GUAGU), which has been extracted from the corresponding NMR structure. The root-mean-square displacement (RMSD) during the simulation was evaluated from the geometric coordinates of all atoms of the complex, as well as from the RBD site with respect to those of the initial structures. As shown in Figure S3A, the RMSD values of the predicted MSI1-RBD1:GUAGU increase up to ~ 5.0 Å during the first 20 ns, then decrease to ~ 3.1 Å with a fluctuation of approximately 0.5 Å until the end of the simulation. For MSI1-RBD2 (Figure S3B), RMSD increase is found within the first 20 ns and maintained at around 6.0 Å with a fluctuation at 1.0 Å up to 100 ns. The RBD site exhibits a much lower RMSD of ~ 1.0–1.7 and ~ 2.0–2.3 Å in both systems, respectively. This implies high fluctuation at the protein terminals, especially at the C terminal end, as well as flexible loops, and the 3' end of the GUAGU pentamer (Figure S3).

To estimate the canonical RNA binding affinity, the SIE method was employed on 200 snapshots taken from the last 20 ns. The Δ*G*_bind_ results of MSI1–RBD1 (− 16.77 ± 0.66 kcal/mol) and MSI1–RBD2 (− 16.54 ± 0.99 kcal/mol) are comparable, and the Coulomb interaction plays a significant role in RNA binding, approximately 2–3 times higher than the vdW interaction (Table 1). The energy contributions of the residues for RNA recognition (Figure S4) show that the 5'-G of GUAGU RBD1 interacts with Trp29 in (black), while RBD2 connects with Asp143. Likewise, U2 interacts with Phe23, Gly26, Phe63, and Lys93 in RBD1, while in RBD2, stabilization is detected by Phe112 and Gly115. The remaining nucleotides of the core MBE, i.e. A3, and G4, are stabilized by a larger number of residues: Phe23, Phe63, Ala95, Phe96, and Arg98 interact with A3 in RBD1, while Phe112, Phe152, Ala184, Gln185, Met190, Pro192, and Thr193 interact with A3 in RBD2. The binding of the three phenylalanines Phe23, Phe63, and Phe65 (RBD1), and Phe112, Phe152, and Phe154 (RBD2) are supported by the experimentally reported NMR structures^13^. The fourth nucleotide, G, is associated with Lys21, Met52, Arg61, Phe65, Phe96, and Arg99 in RBD1, and Lys110, Phe152, Phe154, Gln185, Lys187, Pro192, and Arg199 in RBD2. Finally, a large contribution of the 3'-terminal U is due to the C-terminal residues Pro97, Arg98, Arg99, Gln101, and Pro102 in RBD1, while the terminal U nucleotide flips up and interacts with Met141 and Lys144 in RBD2.

**Table 1:** Binding free energy (kcal/mol) of MSI1 RBD1/2:GUAGU complexes calculated by the solvated interaction energy method (n = 200, SD = standard deviation).

Energy Component (kcal/mol)	RBD1 (± SD)	RBD2 (± SD)
Δ*E*_vdW_	− 99.44 ± 5.50	− 119.58 ± 6.49
Δ*E*_c_	–355.64 ± 15.21	− 250.14 ± 24.47
γΔMSA	− 15.08 ± 0.40	− 18.40 ± 1.22
Δ*G*^R^	337.68 ± 12.82	257.84 ± 21.60
C	− 2.89	
α	0.104758	
^a^Δ*G*_bind_	− 16.77 ± 0.66	− 16.54 ± 0.99

### Molecular dynamics study of MSI1-RBD1/2 with alternative RNA motifs

In addition to studying the MSI1-RBD1/2 in complex with GUAGU, which has been obtained from NMR structures, we set out to explore three alternative RNA pentamers, i.e., GUUGU, GGAGU, and GAUGU, by MD simulations. The overall tightness of the MSI1-RBD1 and MSI1-RBD2 in complex with these four RNA pentamers bound was assessed by radius of gyration (R_g_) in Figure S5. To this end, nucleotides of the pentamer triplet cores were adjusted using the NMR structure to obtain starting geometries for MD. The last snapshots from all simulations were superimposed and are depicted in Figure S6, while the root mean square fluctuation (RMSF) of protein residues is shown in Figure S7. MSI1-RBD1/2 in complex with the canonical RNA GUAGU show the highest stability among all complexes, i.e., the pentanucleotide is well accommodated within the RBD site.

**Figure 3:**
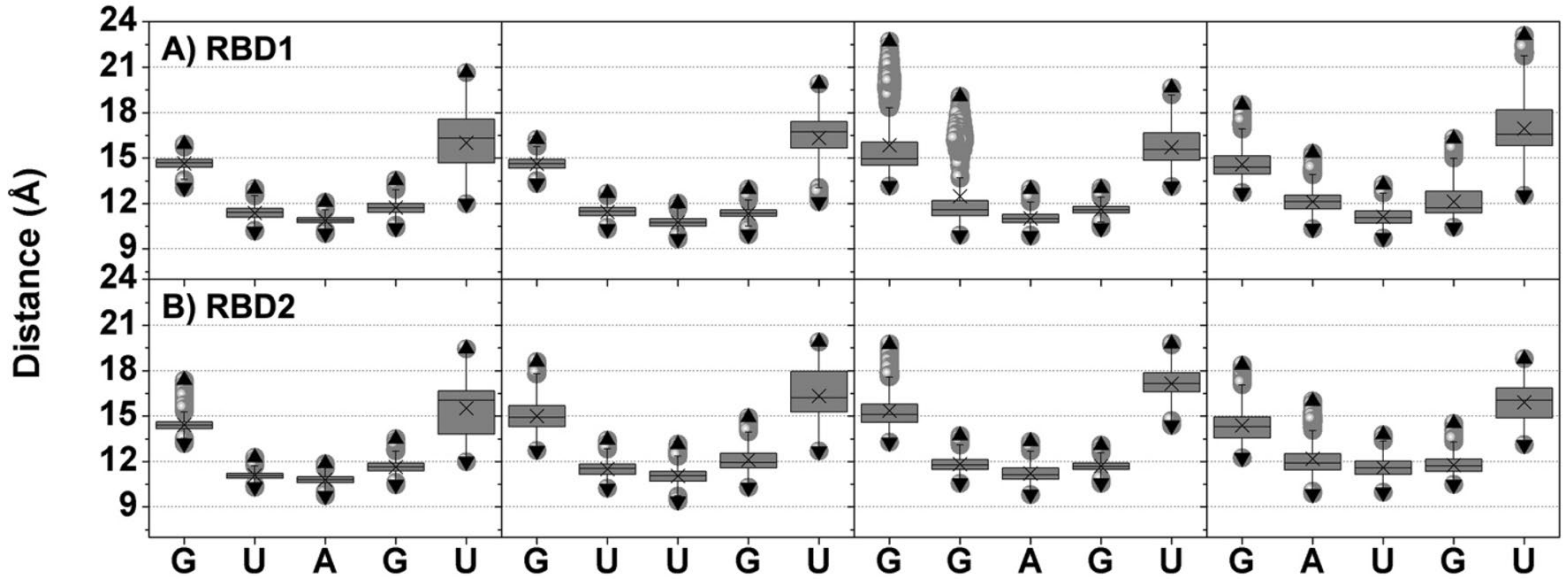
Distances between the centers of mass of each nucleotide and protein in (**A**) MSI1–RBD1 and (**B**) MSI1–RBD2, bound with the four RNAs. Data were taken from the last 20 ns of all five simulations (1000 snapshots in total). Grey boxes cover the area between the 25th and 75th percentiles, while whiskers determine the 5th, and 95th percentiles, respectively. Upward and downward triangles represent maximum and minimum values, respectively. Mean values are indicated by a cross, and outliers are depicted by bullets.

The most considerable difference in pentanucleotide conformation is found in the GAUGU system. As shown in Fig. 3, the central trinucleotides of all models are placed significantly closer to the protein center (distance distribution of ∼10–12 Å) than the flanking nucleotides (∼14–18 Å). The structural fluctuation of the C-terminal (Figures S6 and S7) is related to the high mobility of the RNA 3' end, as seen by large interquartile ranges in Fig. 3. A change from A to U at position 3 (GUUGU) moves the C-terminal closer to the 3' end in RBD1, leading to better stabilization. For GGAGU, the substitution from U to G at the second position results in increased distances of this nucleotide in both RBDs as well as at the 5' end in RBD1 and the two ends in RBD2. Interestingly, changing two nucleotides of the trinucleotide core, leading to GAUGU, results in significantly lengthened distances. Remarkably, the range of the distance distributions is substantially wider in the case of GAUGU compared to the original GUAGU pentamer. By considering the distance plot, the structural fluctuation of RNAs within the RBD1/2 site is ranked in the order of GUUGU<GUAGU<GGAGU<<GAUGU in RBD1; and GUAGU<GGAGU<GUUGU<<GAUGU in RBD2. In other words, RNA motifs with less structural fluctuation show a higher affinity for MSI1-RBD1/2.

**Figure 4:**
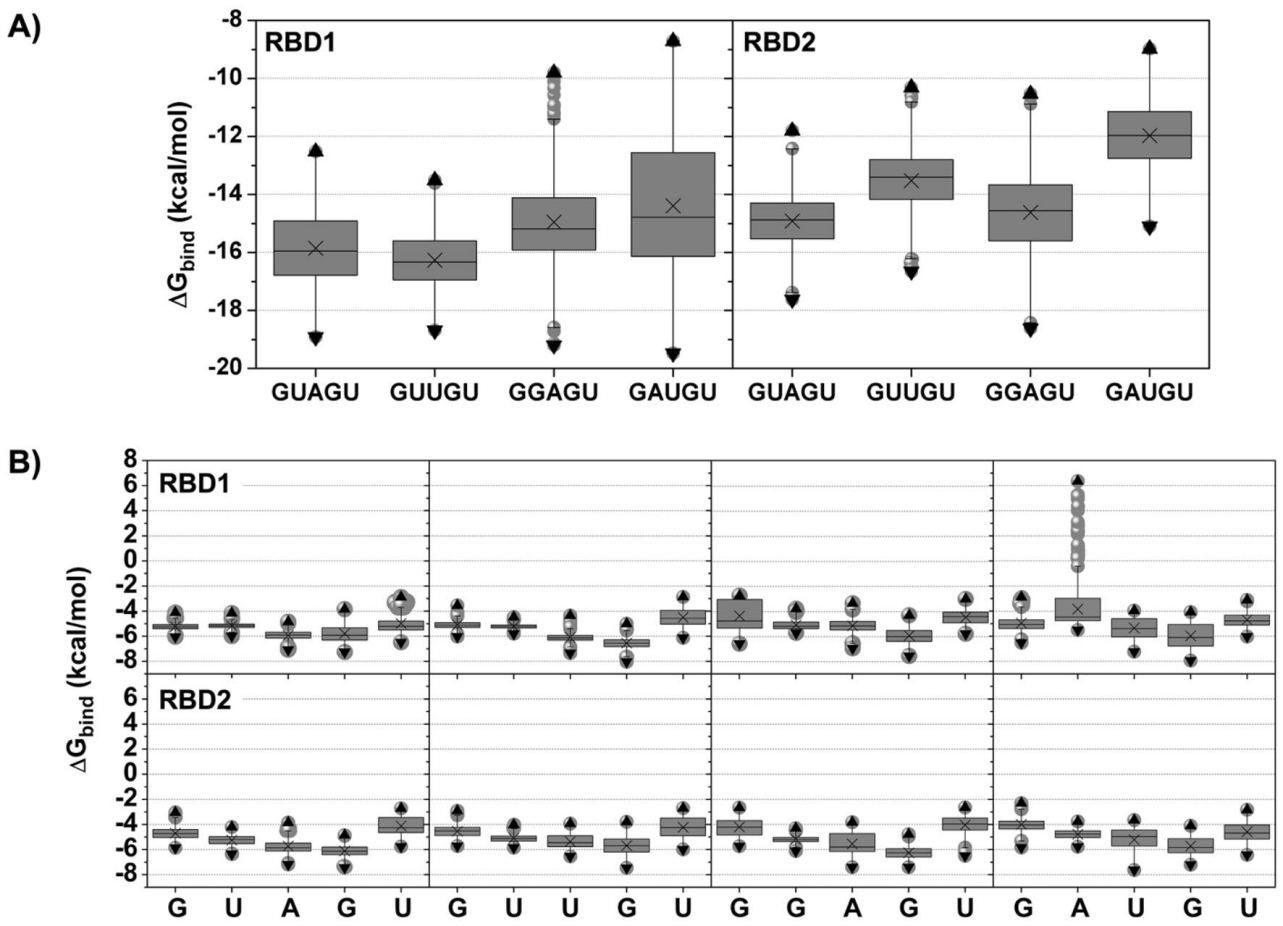
Binding free energies (Δ*G*_bind_) of pentanucleotide (**A**) and individual nucleotide (**B**) binding to MSI1–RBD1/2, calculated by the solvated interaction energy method. Data are taken from the last 20 ns of all five simulations (1000 snapshots in total). Grey boxes cover the area between the 25th and 75th percentiles, with crosses indicating the mean value. Whiskers determine 5th, and 95th percentiles, respectively. Upward and downward triangles represent maximum and minimum values.

The SIE method was applied for Δ*G*_bind_ calculations to predict the pentanucleotide binding strength to MSI1-RBD1/2. From Fig. 4 and Table S1, the Δ*G*_bind_ values of GUAGU, GUUGU, GGAGU, and GAUGU in complex with MSI1-RBD1 are − 15.86 ± 1.22, − 16.27 ± 0.93, − 14.95 ± 1.46, and − 14.39 ± 2.23 kcal/mol, respectively. The overall binding affinity is relatively lower in the case of MSI1-RBD2, i.e., they are − 14.92 ± 0.91, − 13.53 ± 1.07, − 14.62 ± 1.42, and − 11.97 ± 1.13 kcal/mol. The energy components of MSI1-RBD1:GUAGU are comparable to the predicted model (Table 1), while the decreased Coulomb interaction (~ 2-fold) in MSI1-RBD2 is compensated by the reduction in the change of the reaction energy upon binding (2-fold). Although the resulting Δ*G*_bind_ follows the same trend as the structural data above, RNA–protein interactions must be taken into consideration for RNA recognition by a specific protein. From this perspective, the binding of each nucleotide was evaluated by using the SIE binding free energy and MM/PBSA per-residue decomposition free energy calculations.

The highest binding affinity of GUUGU to RBD1 in Fig. 4A can be explained by a strong binding of the central trinucleotide UUG of − 5.22 ± 0.18, − 6.09 ± 0.35, and − 6.56 ± 0.42 kcal/mol (Fig. 4B and Table S2). The trinucleotide binding is slightly weaker in GUAGU. The binding free energies of the remaining pentanucleotides GGAGU and GAUGU are significantly weaker, as can also be seen from the individual nucleotide contributions. In the case of RBD2, the GUAGU pentamer has the lowest binding free energy, whose trinucleotide binding free energies are − 5.24 ± 0.40, − 5.71 ± 0.65, and − 6.11 ± 0.48 kcal/mol, respectively. According to the total energy contributions (Fig. 4A), the other pentanucleotides exhibit a substantially weaker binding.

**Figure 5:**
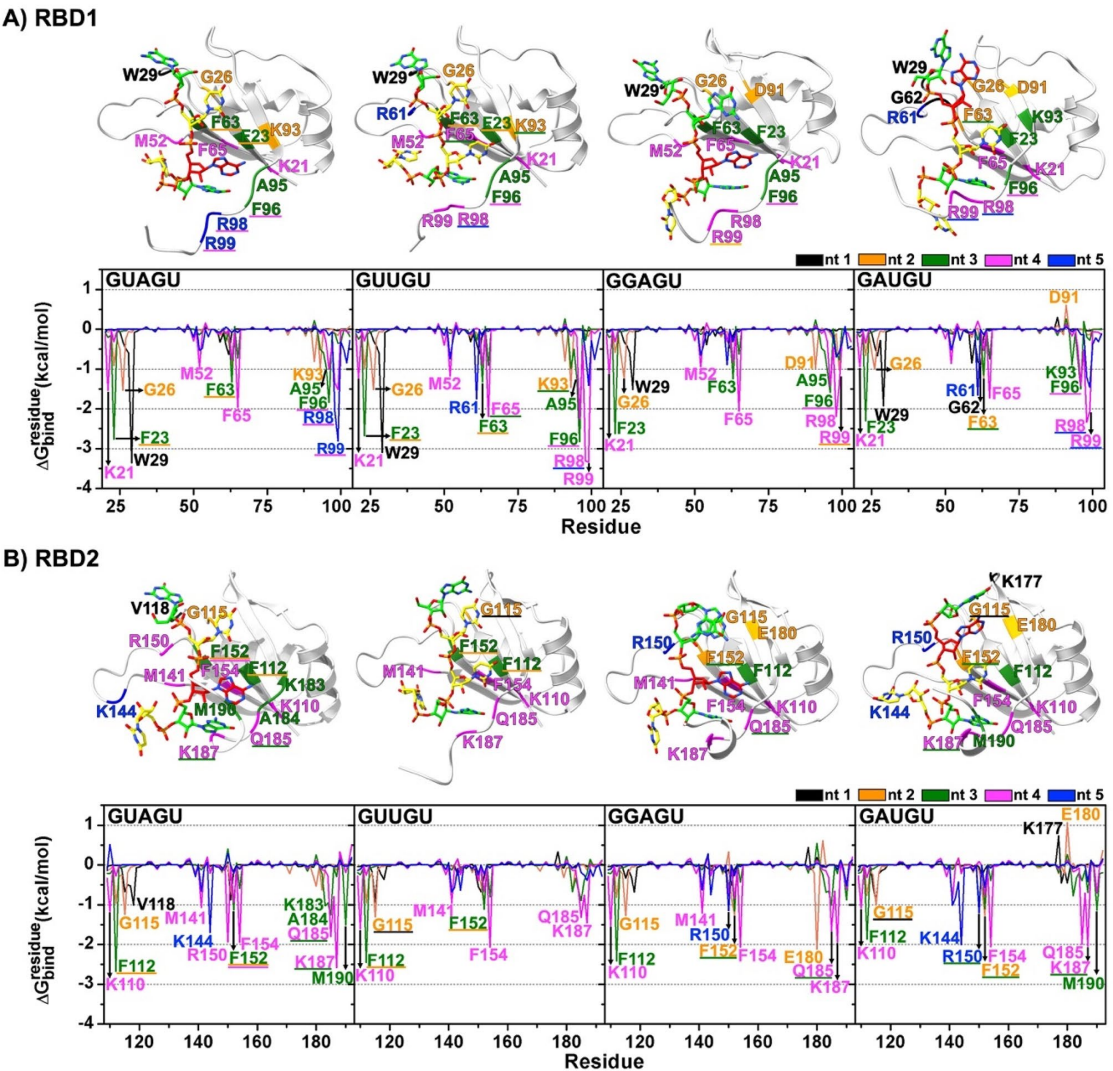
Per–residue binding free energy contribution (${\Updelta{\text{G}}}_{\text{bind}}^{\text{residue}}$) for the five nucleotides (nt1–nt5) of (**A**) MSI1–RBD1:RNAs and (**B**) MSI1–RBD2:RNAs, derived from the average of 1000 snapshots of the last 20 ns of GUAGU, GUUGU, GGAGU and GAUGU, respectively. Residues with ${\Updelta{\text{G}}}_{\text{bind}}^{\text{residue}}$) ≤  − 0.90 kcal/mol and ≥ 0.60 kcal/mol are labeled. Residues that interact with two nucleotides are underlined.

Figure 5 shows the residue contributions for each nucleotide binding MSI1–RBD1 and RBD2. Negative and positive Δ*G*_bind,res_ values represent the nucleotide stabilization and destabilization, respectively. Most RNA-interacting residues are found on the beta-sheet face; However, certain residues in the flexible loop regions also interact with RNA. For RBD1 (Fig. 5A), the G at the 5' end interacts with Trp29 in all models (black). The U at position 2 of the GUAGU and GUUGU pentamers has interactions with Phe23, Gly26, Phe63, and Lys93, while the G2 of GGAGU binds with Gly26, Asp91, and Arg99. The situation is different for GAUGU. Although the A is stabilized by Gly26 and Phe63, it is destabilized by Asp91, which is in agreement with our binding free energy data (Fig. 4B). The central nucleotide (position 3) of GUAGU, GUUGU and GGAGU interacts with Ala95 and the three phenylalanines Phe23, Phe63, and Phe96^13^. The energy contribution of Ala95 is reduced for the central nucleotide of GAUGU. Among all RNAs, the positively charged residues Arg98 and Arg99 provide the highest stabilization to G4 of GUUGU, relating to its highest binding affinity (Fig. 4B). Additionally, Lys21, Met52, and Phe65 are also important for the binding of this nucleotide. Their contributions are lowered in the GAUGU model. At the 3' end, we observe stabilization from positively charged residues at the C-terminal: Arg98 and Arg99 in GUAGU; Arg61 and Arg98 in GUUGU; and Arg61, Arg98 and Arg99 in GAUGU. These contributions are substantially lower in GGAGU.

For RBD2 the 5' G of the GUAGU pentanucleotide interacts with Val118, which is located at a structurally similar position as Trp29 in RBD1. The second nucleotide of all pentamers has a weak interaction with Gly115 and Phe152, while the third nucleotide of all pentamers interacts with Phe112 and Phe152, which correspond to residues as Phe23 and Phe63 in RBD1. The fourth nucleotide of all pentamers interacts with Lys110, Phe154, Gln185, and Lys187. For GAUGU, we observed a repulsive interaction between the 5'-terminal G and Lys177, and A2 and Glu180, thus highlighting the poor interaction of GAUGU with MSI1-RBD2 (Fig. 5B). Furthermore, at protein–RNA interfaces, stacks can be intermolecular, formed by rings of the nucleic acid bases with the aromatic side-chains of phenylalanine, arginine, alanine, lysine, glycine, and methionine. However, stacking interactions do not appear to provide significant sequence specificity in protein–RNA complexes (Figure S8).

**Figure 6:**
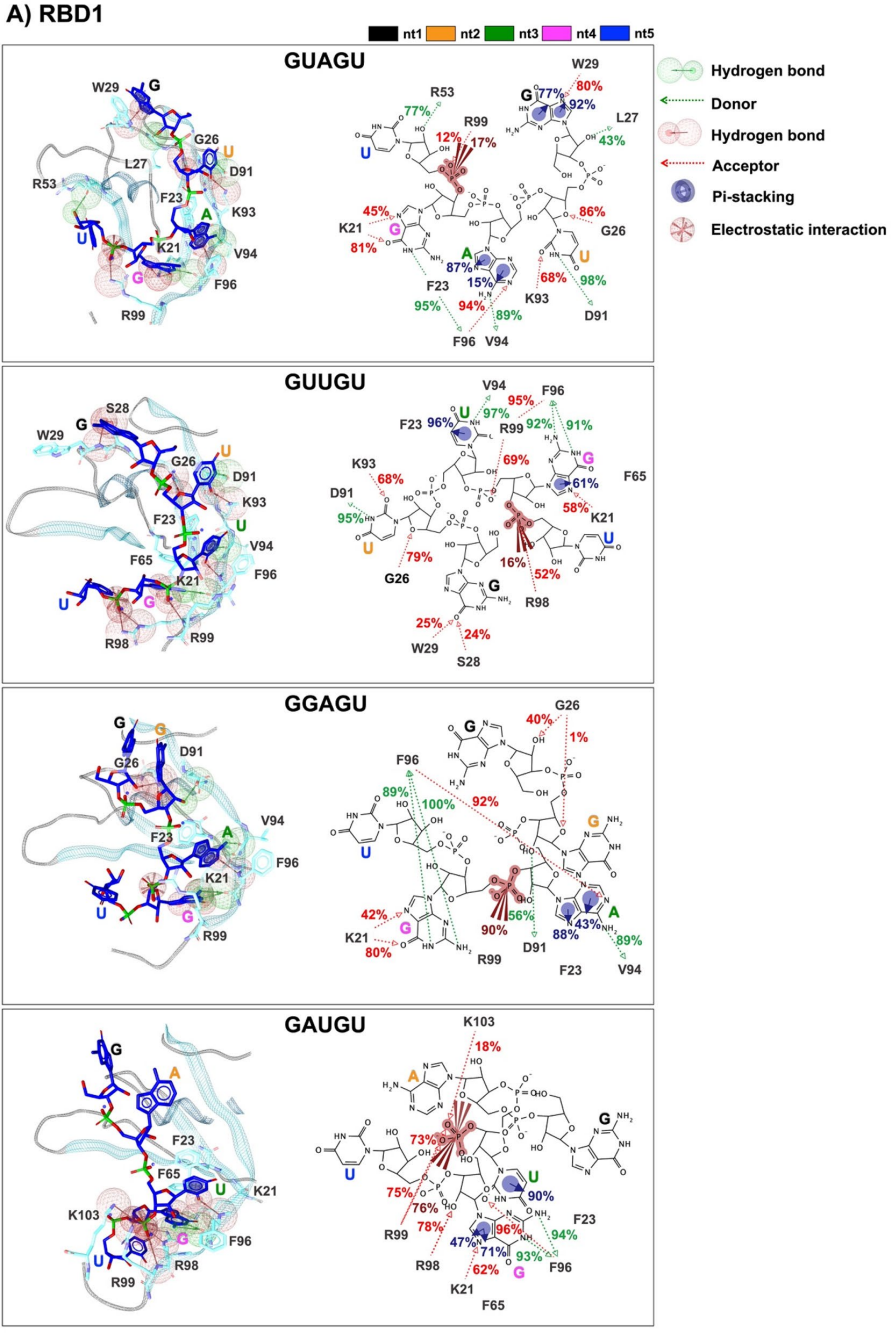

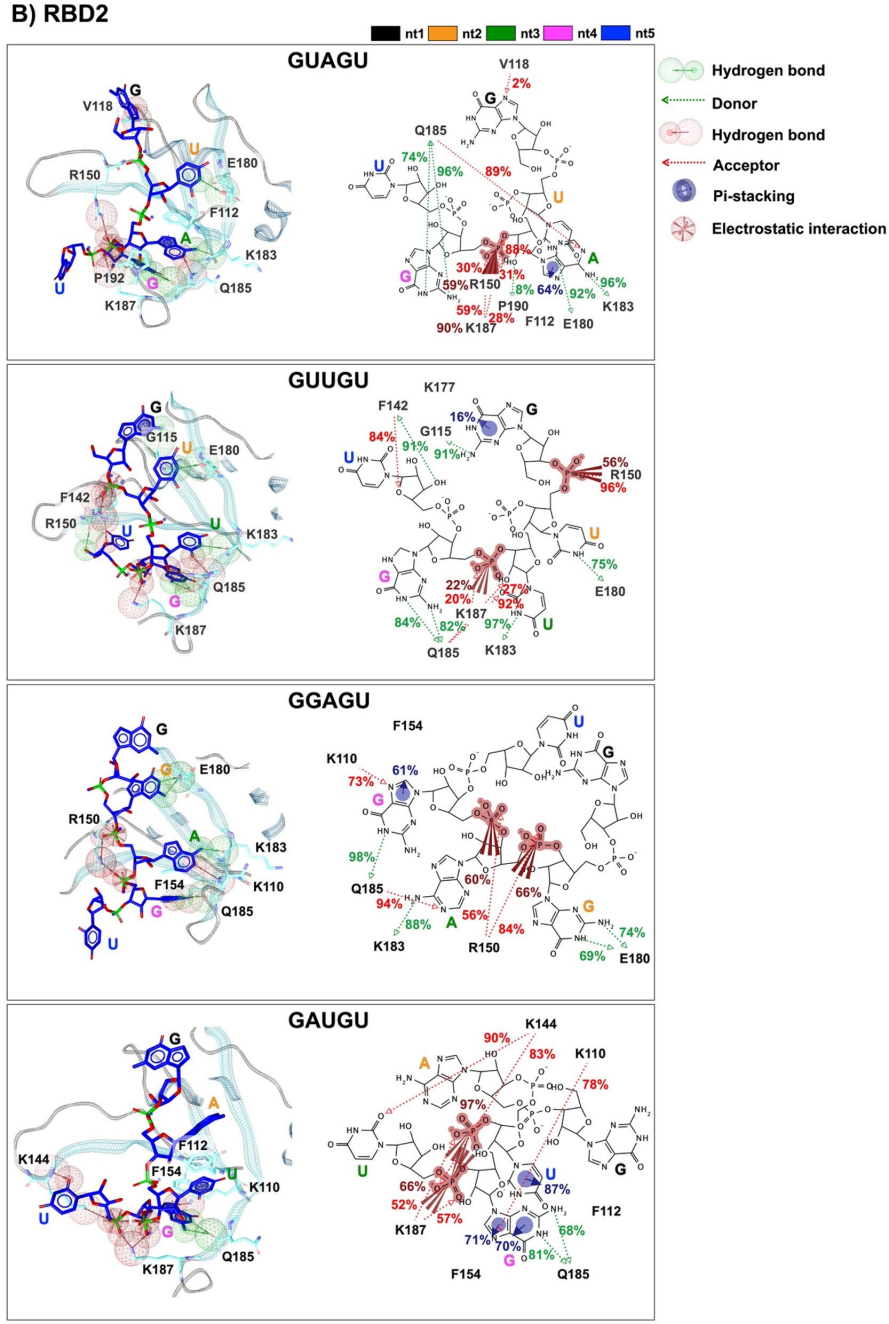
Pharmacophore models and protein–RNA interaction maps with percentage of occurrence in the last 20 ns of (**A**) MSI1–RBD1 and (**B**) MSI1–RBD2 in complex with the four RNA pentamers GUAGU, GUUGU, GGAGU, and GAUGU. Pi–pi interactions are highlighted by blue circles, electrostatic interactions are highlighted by red spheres, and hydrogen bond properties are highlighted by green/red spheres and vectors.

The protein–RNAs interaction map with percentage of occurrence from the last 20 ns is depicted in Figs. 6A and B. In the MSI1–RBD1 model, the hydrogen bond donor (HBD) features were discovered in multiple interactions with the protein, whereas nitrogen and oxygen atoms in the aromatic rings of GUAGU interacted with Leu27 (43%), Arg53 (77%), Asp91 (98%), Val94 (89%),  and Phe96 (95%). On the other hand, the most hydrogen bond acceptor (HBA) formation was observed in the GUUGU model, as shown by a red line in the position of Lys21 (58%), Gly26 (79%), Ser28 (24%), Trp29 (25%), Lys93 (68%), Phe96 (95%), Arg98 (52%) and Arg99 (69%), which are bound to the nitrogen and oxygen atoms of the aromatic rings. Pi–pi stacking was found in a variety of links with the protein, such as Phe23 (87%, 96%, 43–88%, and 90% in GUAGU, GUUGU, GGAGU, and GAUGU model, respectively), Trp29 (77–92% in GUAGU model), Phe65 (61% and 71% in GUUGU, and GAUGU model, respectively), Phe96 (15% in GUAGU model) and Arg98 (47% in GAUGU model). Electrostatic interactions were also discovered in the phosphate group between the fourth and fifth units of the RNA in the GUAGU (G4 and U5) and GUUGU (G4 and U5) models, whereas in GGAGU and GAUGU they were found in three to fourth (A3 and G4) and two to three (A2 and U3) units, respectively.

HBD features were discovered in several interactions with the protein in the MSI1–RBD2 model, where nitrogen and oxygen atoms in the aromatic rings interact with Gly115, Phe142, Glu180, Lys183, Gln185, and Pro190. On the other hand, HBA formation was observed in Lys110, Val118, Phe142, Lys144, Arg150, Gln185, and Lys187. Pi–pi interactions were discovered in Phe112 (64%) in the GUAGU model, Lys177 (16%) in the GUUGU model, Phe154 (61%) in the GGAGU model, Phe112 (87%), and Phe154 (70–71%) in the GAUGU model. One electrostatic interaction was also discovered for the phosphate group between the third and fourth unit of the RNA in the GUAGU, whereas two electrostatic interactions were found in the GUUGU, GGAGU, and GAUGU.

## Discussion

Musashi genes have attracted considerable interest as regulators of stem and progenitor cell characteristics. In the present study, we evaluated the three-dimensional structures of the MSI1 in complex with RNA. To this end, we studied the association of MSI1 RNA-binding domains 1 and 2 (RBD1 and RBD2) with different RNA motifs. We investigated the canonical RNA motif GUAGU, as well as the alternative motifs GUUGU (good binding affinity), GGAGU (weaker binding affinity), and GAUGU (unfavorable binding affinity). We compared the protein structures without RNA from the PDB with Alphafold2-predicted geometries and found that protein structures of the binding domains are highly similar, therefore no conformational changes on the protein occur upon binding of the RNA. In addition, our results corroborate earlier findings that MSI1 RBD1 and RBD2 structures are remarkably similar, despite variation in the underlying primary sequence^21^. To investigate the properties of the RNA–protein association complexes, we performed molecular dynamics simulations and computed the interaction energies by the SIE method.

In agreement with earlier results^17^, the central trinucleotides of the RNA pentamers (Musashi binding element, MBE) are more rigid than the flanking nucleotides. Moreover, the flanking nucleotides lack interaction with MSI1 RBDs^13^, suggesting that MSI1–RBD1 and RBD2 require the central trinucleotides for recognition. Our MD simulations show that the central trinucleotides of the RNA motifs exhibit a significantly lower distance to the MSI1 RBDs than the enclosing nucleotides. Thus, the central trinucleotides play an important role in the interaction of MSI1–RBD1 and RBD2 with RNA.

We identified key residues for MSI1–RBD1 binding, specifically Phe23, Trp29, Phe63, Phe65, Phe96, Arg98 and Arg99 interacting with nucleotides. Our MD simulations are consistent with the fact that Phe23, Phe63 and Phe65 are conserved among all models and interact with A3 and G4 of the pentanucleotides. For MSI1–RBD2, Lys110, Phe112 Gly115, Phe152, Phe154, Gln185, and Lys187 are in contact with the nucleotides. Stacking interactions between evolutionarily conserved phenylalanine (Phe23:RBD1 and Phe112:RBD2, Phe63:RBD1 and Phe152:RBD2, Phe65:RBD1 and Phe154:RBD2) and non-conserved residue tryptophan (Trp29:RBD1 and Val118:RBD2), phenylalanine (Phe96:RBD1 and Gln185:RBD2) of MSI1 and the aromatic bases and ribose rings of the RNA contribute to target recognition within MSI1.

The SIE calculations lead to the following conclusions: Assessment of the contributions to the overall binding free energy of individual nucleotides of the GUAGU and GGAGU motifs shows that the central core nucleotides have the largest interaction energies, with A3 and G4 nucleotides exhibiting the most pronounced contribution. The flanking nucleotides contribute significantly less. Our calculations show that for RBD1, the GUUGU motif possesses the largest binding free energy, followed by GUAGU. While this appears counterintuitive, it is in line with earlier data that assessed opening energy z scores at the level of RNA secondary structures. RBD2 on the other side has overall smaller interaction energy, with the GUAGU motif showing the highest binding affinity for all pentamers.

Calculated decomposition energies clearly show the contributions of individual amino acids to the complexation of the RNA. For RBD1, we should highlight Phe23, Phe63 and Phe65 because of their substantial interaction with the core motif. In analogy, Phe112, Phe152 and Phe154 of RBD2 show a strong interaction with the core trinucleotides.

In summary, we show here the feasibility of MD and SIE calculations to investigate the selectivity of RNA–protein interaction complexation. Further studies are warranted, such as the binding of a longer RNA chain that includes both binding motifs of the two RBDs of Musashi proteins. MSI1 plays a particularly important role in brain development, and increased expression of Musashi proteins in patients infected with Zika virus during pregnancy has been associated with microcephaly. A better understanding of the interaction of the MSI proteins with RNA, in particular, Zika virus RNA is required to address the issue of inhibiting Zika virus replication in infected patients without affecting brain development.

## Supplementary Information

Supplementary Information.

## Data Availability

The datasets used and/or analyzed during the current study are available from the corresponding author on reasonable request.
