# Supplementary material for: Theoretical studies on RNA recognition by Musashi 1 RNA-binding protein
Source: Sci Rep. 2022 Jul 15;12:12137. doi: 10.1038/s41598-022-16252-w (PMC9287312; doi:10.1038/s41598-022-16252-w)
Supplement: Supplementary file 1 — Supplementary Information. [file 41598_2022_16252_MOESM1_ESM.docx]

**Supplementary File**

**Theoretical studies on RNA recognition by Musashi 1 RNA–binding protein**

Nitchakan Darai^1^, Panupong Mahalapbutr^2^, Peter Wolschann^3^, Vannajan Sanghiran Lee^4^, Michael T. Wolﬁnger^3,5*^ and Thanyada Rungrotmongkol^1,6*^

^1^Program in Bioinformatics and Computational Biology, Graduate School, Chulalongkorn University, Bangkok, 10330, Thailand
^2^Department of Biochemistry, Faculty of Medicine, Khon Kaen University, Khon Kaen, 40002, Thailand

^3^Department of Theoretical Chemistry, University of Vienna, Währinger Strasse 17, Vienna, 1090, Austria

^4^Department of Chemistry, Faculty of Science, University of Malaya, Kuala Lumpur, 50603, Malaysia

^5^Research Group Bioinformatics and Computational Biology, Faculty of Computer Science, University of Vienna, Währinger Strasse 29, Vienna, 1090, Austria

^6^Center of Excellence in Biocatalyst and Sustainable Biotechnology, Faculty of Science, Chulalongkorn University, Bangkok, 10330, Thailand
*Corresponding authors. MW Fax: +43 1–4277–879320; Tel: +43 1–4277–52747, TR Fax: +66 2 218–5418; Tel: +66 2 218–5426

**E–mail address:** michael.wolfinger@univie.ac.at (M.Wolﬁnger), thanyada.r@chula.ac.th (T. Rungrotmongkol)

**Table of Contents**

Table S1................................................................................................................................. 3

Table S2................................................................................................................................. 4

Figure S1................................................................................................................................ 4

Figure S2................................................................................................................................ 5

Figure S3................................................................................................................................ 6

Figure S4................................................................................................................................ 7

Figure S5................................................................................................................................ 8

Figure S6................................................................................................................................ 9

Figure S7................................................................................................................................ 10

Figure S8................................................................................................................................ 11

**Table S1:** Binding free energies (kcal/mol), including standard deviations, of MSI1–RBD1/2 and RNA pentanucleotides, calculated by the solvated interaction energy (SIE) method (n= 1,000, SD=standard deviation).

| **Energy Component (kcal/mol)** | **RBD1** | | | |
| --- | --- | --- | --- | --- |
|  | **GUAGU (±SD)** | **GUUGU (±SD)** | **GGAGU (±SD)** | **GAUGU (±SD)** |
| Δ*E*_vdW_ | –98.73 ± 8.72 | –99.75 ± 6.60 | –93.83 ± 12.95 | –85.06 ± 14.03 |
| Δ*E*_c_ | –317.63 ± 28.72 | –321.63 ± 28.72 | –317.77 ± 22.52 | –312.48 ± 44.98 |
| γΔMSA | –14.51 ± 1.10 | –14.52 ± 0.96 | –14.20 ± 1.79 | –12.61 ± 1.96 |
| Δ*G*^R^ | 307.32 ± 20.79 | 308.20 ± 25.86 | 310.64 ± 19.61 | 304.49 ± 37.98 |
| C | –2.89 | | | |
| α | 0.104758 | | | |
| ^a^Δ*G*_bind_ | –15.86 ± 1.22 | –16.27 ± 0.93 | –14.95 ± 1.46 | –14.39 ± 2.23 |
| **Energy Component (kcal/mol)** | **RBD2** | | | |
|  | **GUAGU (±SD)** | **GUUGU (±SD)** | **GGAGU (±SD)** | **GAUGU (±SD)** |
| Δ*E*_vdW_ | –98.95 ± 7.60 | –87.27 ± 8.34 | –88.99 ± 12.17 | –80.94 ± 8.94 |
| Δ*E*_c_ | –126.27 ± 25.42 | –95.96 ± 20.88 | –108.08 ± 29.07 | –125.24 ± 27.59 |
| γΔMSA | –14.84 ± 0.87 | –12.82 ± 1.38 | –13.55 ± 1.76 | –12.44 ± 1.21 |
| Δ*G*^R^ | 125.27 ± 23.17 | 94.51 ± 18.64 | 98.67 ± 29.86 | 132.56 ± 25.07 |
| C | –2.89 | | | |
| α | 0.104758 | | | |
| ^a^Δ*G*_bind_ | –14.92 ± 0.91 | –13.53 ± 1.07 | –14.62 ± 1.42 | –11.97 ± 1.13 |

^1^The binding free energy (Δ*G*_bind_) computed by Δ*E*_vdW_ and Δ*E*_c_ are the van der Waals interaction and Coulomb interaction, respectively. γΔMSA relates to the change of the molecular surface area are induced by RNA binding. Δ*G*^R^ indicates the change of the reaction energy upon binding and is calculated by solving the Poisson equation with the boundary element method.

**Table S2:** The binding free energy (kcal/mol) of pentanucleotide and each nucleotide binding to MSI1–RBD1/2 calculated with the solvated interaction energy method (n=1,000, SD=standard deviation).

|  |  | **nt1 (±SD)** | **nt2** **(±SD)** | **nt3 (±SD)** | **nt4 (±SD)** | **nt5 (±SD)** |
| --- | --- | --- | --- | --- | --- | --- |
| **RBD1** | **GUAGU** | –5.23 ± 0.26 | –5.17 ± 0.21 | –5.92 ± 0.36 | –5.78 ± 0.69 | –4.97 ± 0.81 |
|  | **GUUGU** | –5.09 ± 0.31 | –5.22 ± 0.18 | –6.09 ± 0.35 | –6.56 ± 0.42 | –4.49 ± 0.69 |
|  | **GGAGU** | –4.35 ± 1.17 | –5.07 ± 0.38 | –5.14 ± 0.52 | –5.92 ± 0.61 | –4.45 ± 0.55 |
|  | **GAUGU** | –4.95 ± 0.65 | –3.85 ± 1.47 | –5.35 ± 0.80 | –5.97 ± 0.93 | –4.69 ± 0.53 |
| **RBD2** | **GUAGU** | –4.69 ± 0.49 | –5.24 ± 0.40 | –5.71 ± 0.65 | –6.11 ± 0.48 | –4.12 ± 0.70 |
|  | **GUUGU** | –4.54 ± 0.44 | –5.10 ± 0.30 | –5.36 ± 0.54 | –5.69 ± 0.66 | –4.22 ± 0.79 |
|  | **GGAGU** | –4.19 ± 0.76 | –5.17 ± 0.27 | –5.55 ± 0.78 | –6.26 ± 0.46 | –4.05 ± 0.72 |
|  | **GAUGU** | –3.98 ± 0.62 | –4.79 ± 0.35 | –5.20 ± 0.94 | –5.71 ± 0.66 | –4.59 ± 0.75 |

**
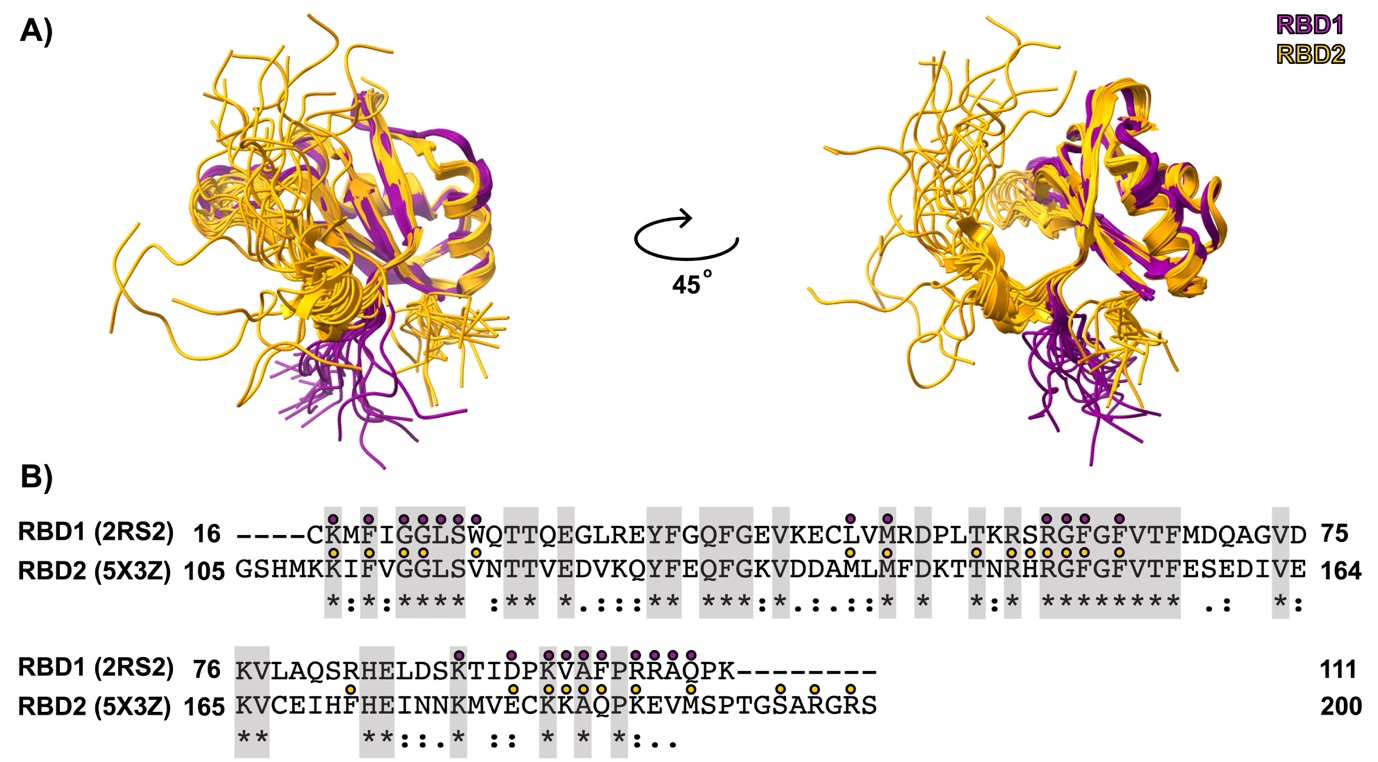
**

**Figure S1:** (**A**) **S**uperimposed NMR structures of MSI1 RBD1 (purple) and MSI1 RBD2 (orange). (**B**) Clustal Omega (1.2.4) multiple sequence alignment and comparison of MSI1 RBD1 (2RS2) and MSI1 RBD2 (5X3Z). Purple and Orange circles highlight residues that interact with RNA in MSI1–RBD1 and MSI1–RBD2.


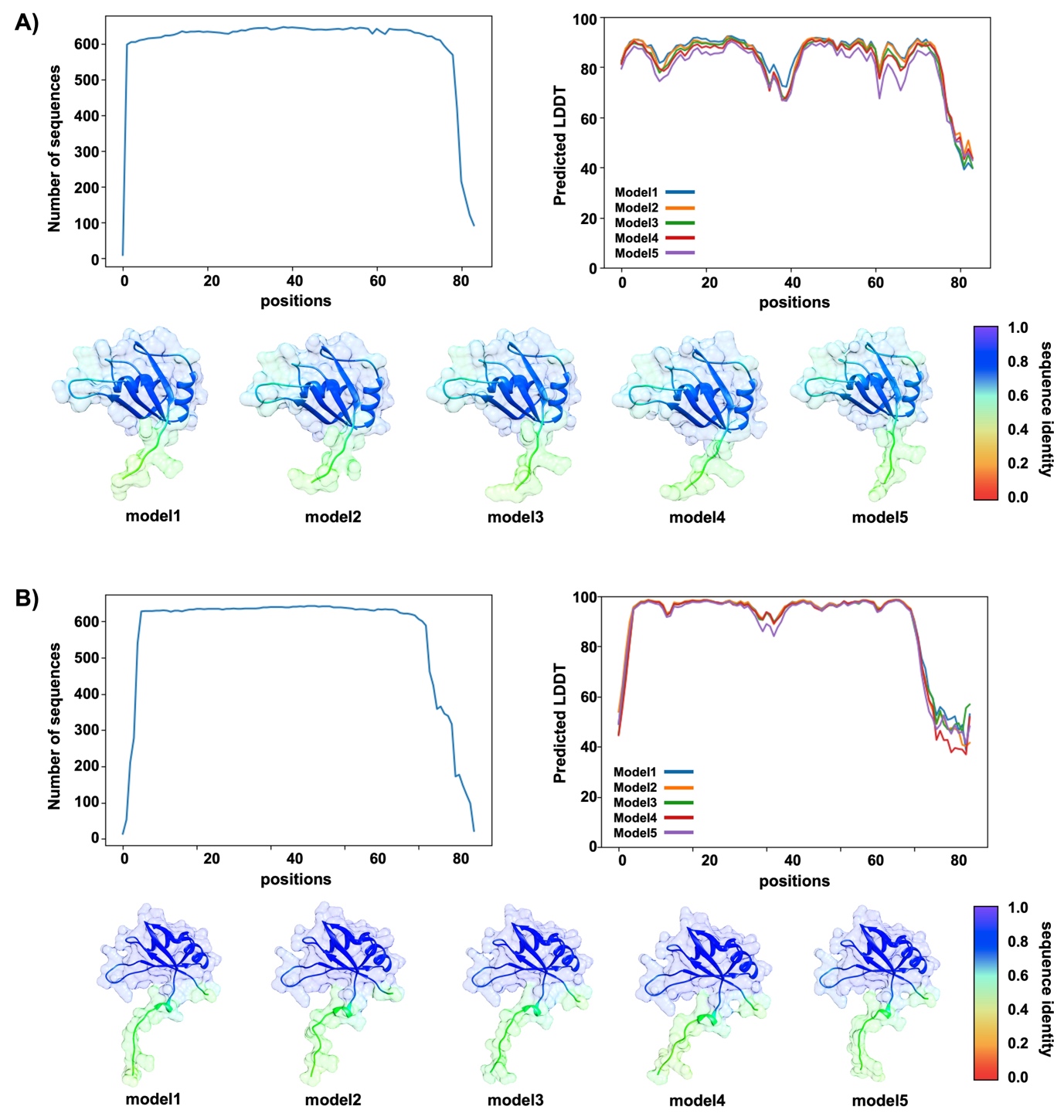


**Figure S2:** (**A**) The number of sequences per position, AlphaFold2 confidence measures (pLDDT) and all five AlphaFold2 models of MSI1–RBD1. (**B**) The number of sequences per position, AlphaFold2 confidence measures (pLDDT) and all five AlphaFold2 models of MSI1–RBD2.


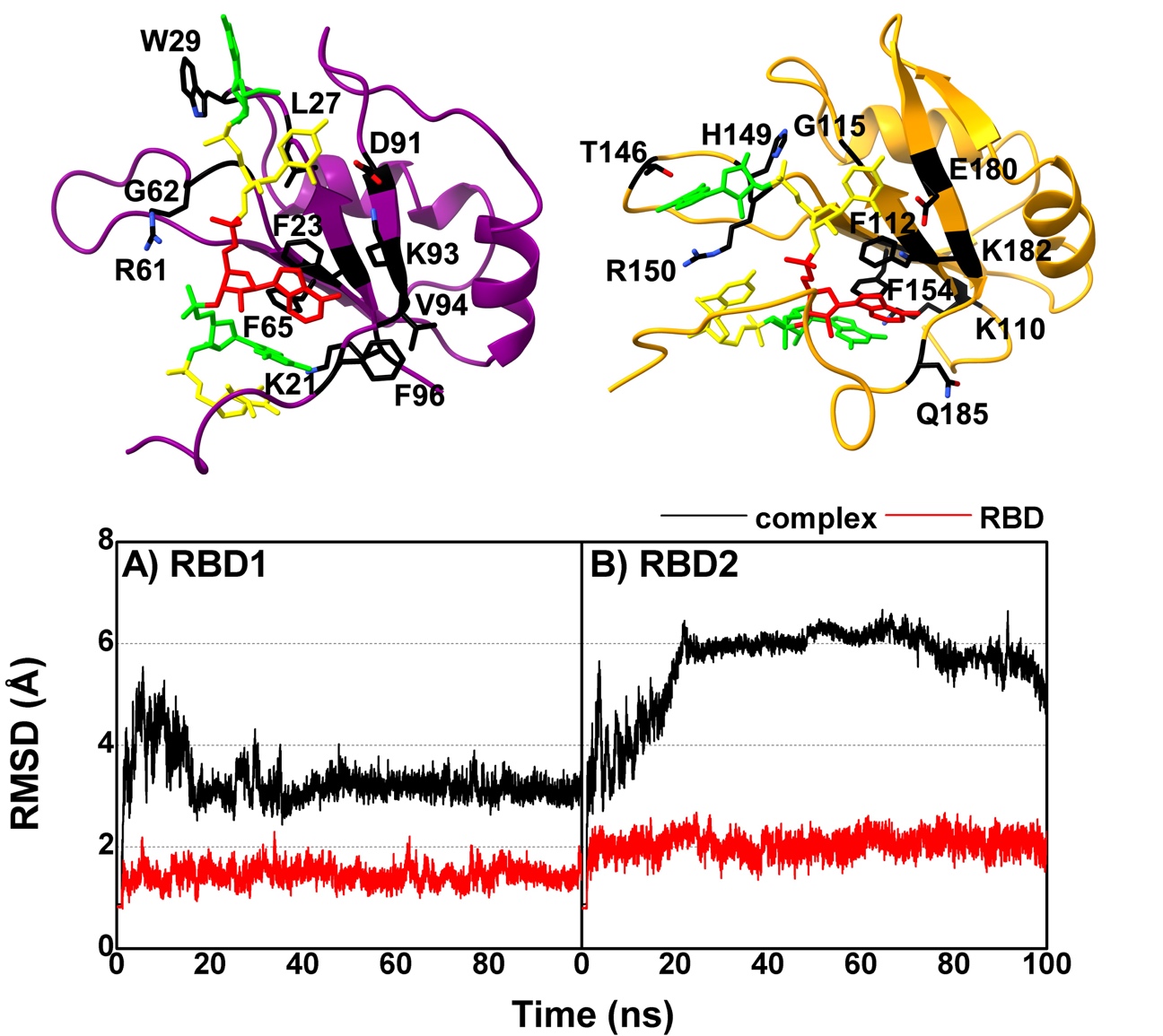


**Figure S3:** All–atom RMSD plots for the predicted model1/GUAGU complexes: (**A**) MSI1–RBD1 and (**B**) MSI1–RBD2. The residues in RBD site Lys21, Phe23, Leu27, Trp29, Arg61, Gly62, Phe65, Asp91, Lys93, Val94, and Phe96 for RBD1; and Lys110, Phe112, Gly115, Thr146, His149, Arg150, Phe154, Glu180, Lys182, and Gln185 for RBD2 are shown in black^13,14^.


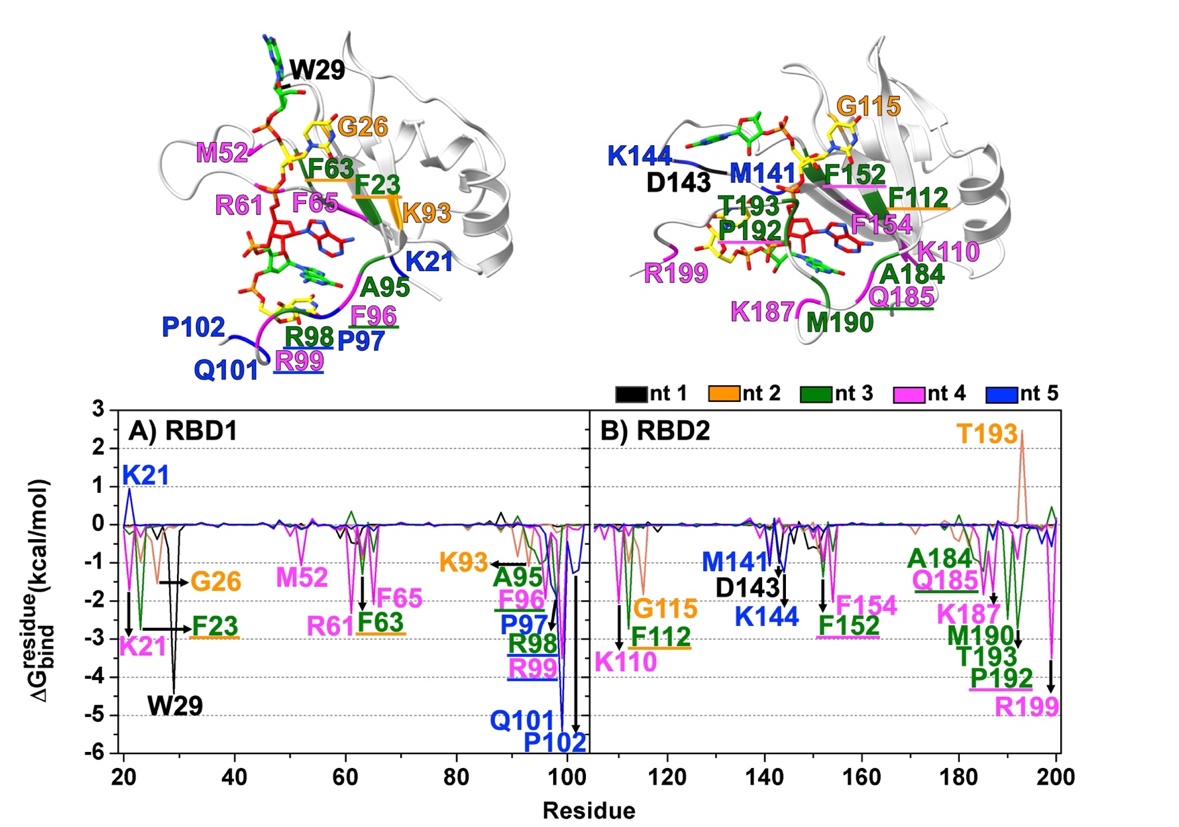


**Figure S4:** Per–residue binding free energy contribution (${\text{Δ}\text{G}}_{\text{bind}}^{\text{residue}}$) for the five nucleotides (nt1–nt5) of (**A**) MSI1–RBD1:GUAGU and (**B**) MSI1–RBD2:GUAGU derived from the last 20 ns. Residues with ${\text{Δ}\text{G}}_{\text{bind}}^{\text{residue}}$ ≤ −0.9 kcal/mol and ≥ 0.6 kcal/mol are labeled. Residues interacting with particular nucleotides are color–coded, as given in the figure. Protein residues that interact with two nucleotides are underlined.


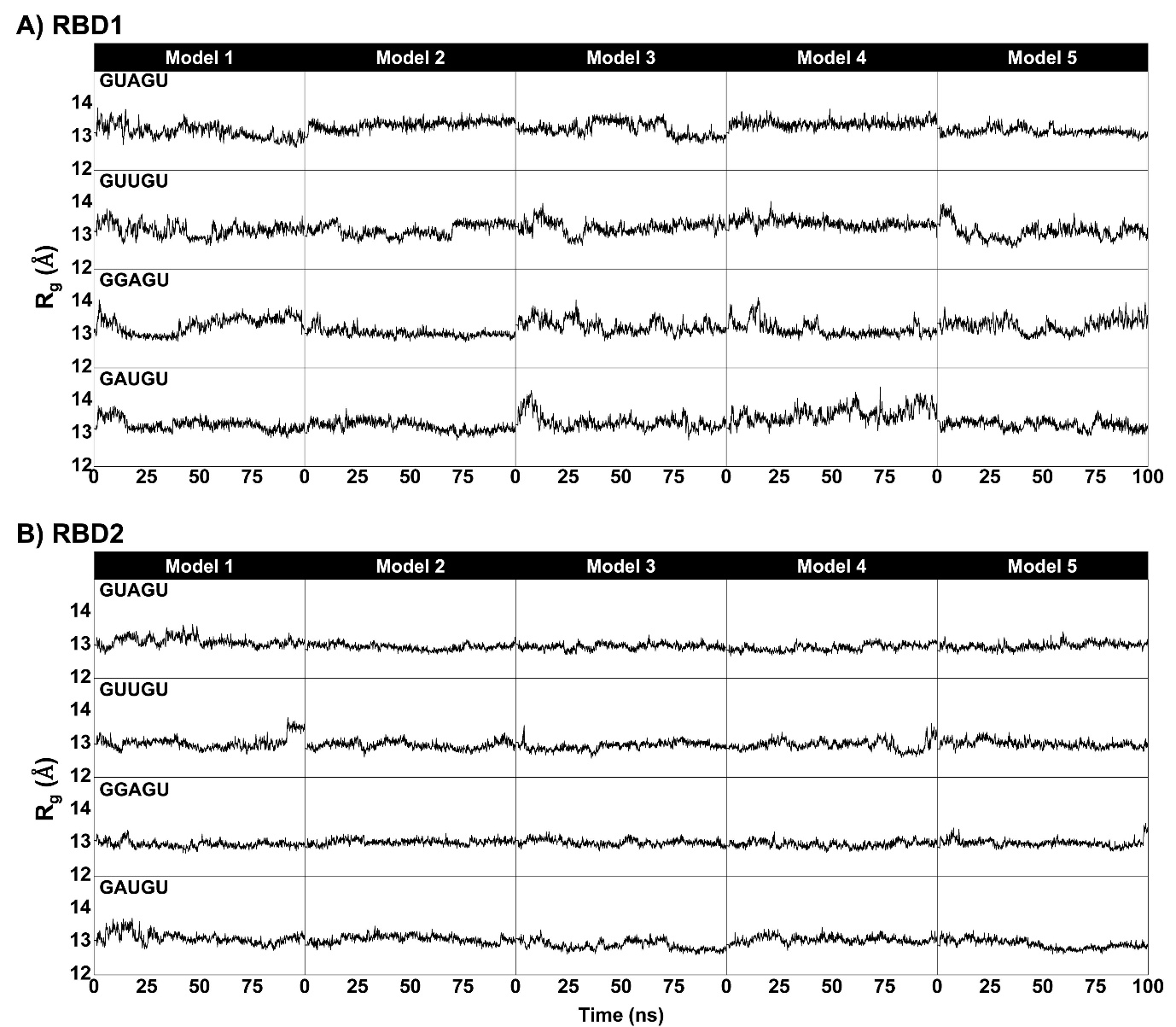


**Figure S5:** Radius of gyration (R_g_) of (**A**) MSI1–RBD1 and (**B**) MSI1–RBD2 in complex with the four RNA pentamers GUAGU, GUUGU, GGAGU, and GAUGU, plotted along with the simulation time.


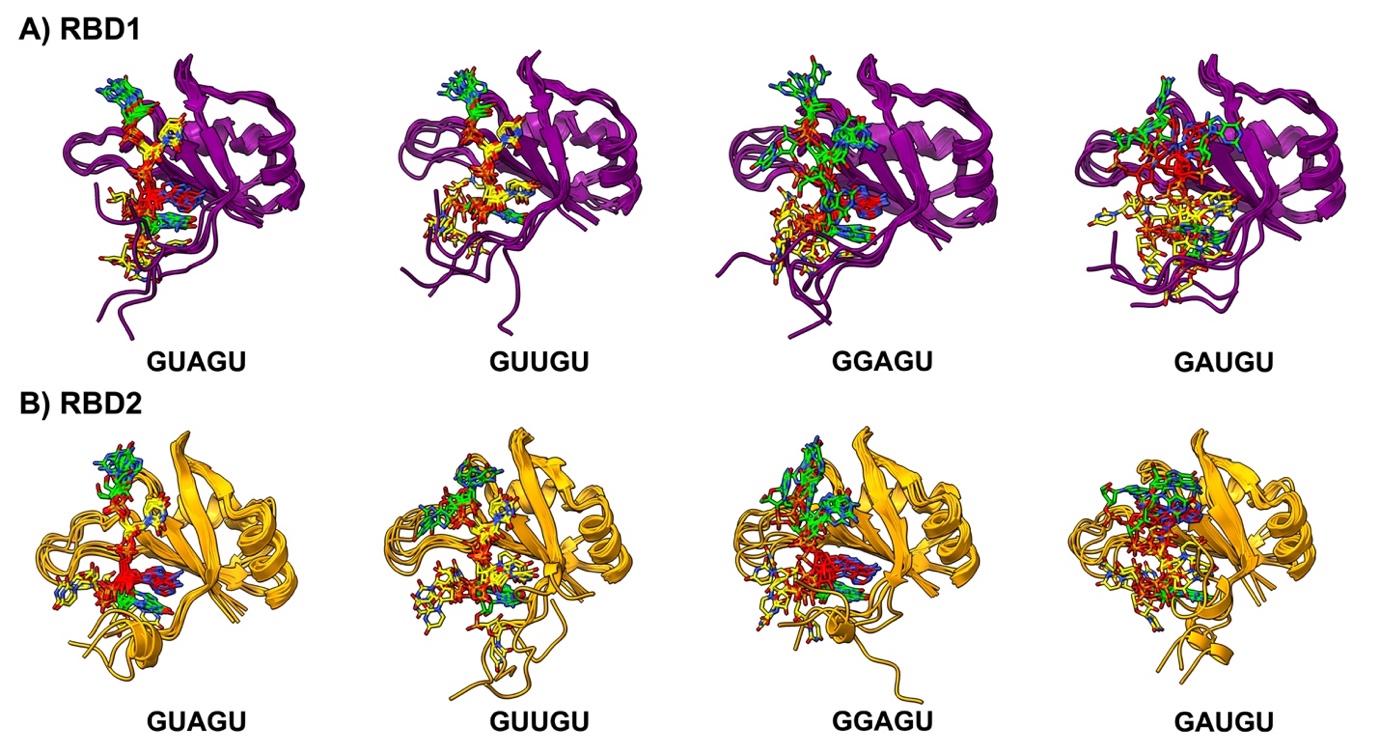


**Figure S6:** Superimposition between the last MD snapshots taken from five individual simulations of (**A**) MSI1–RBD1 and (**B**) MSI1–RBD2 in complex with the four RNA pentamers GUAGU, GUUGU, GGAGU, and GAUGU.


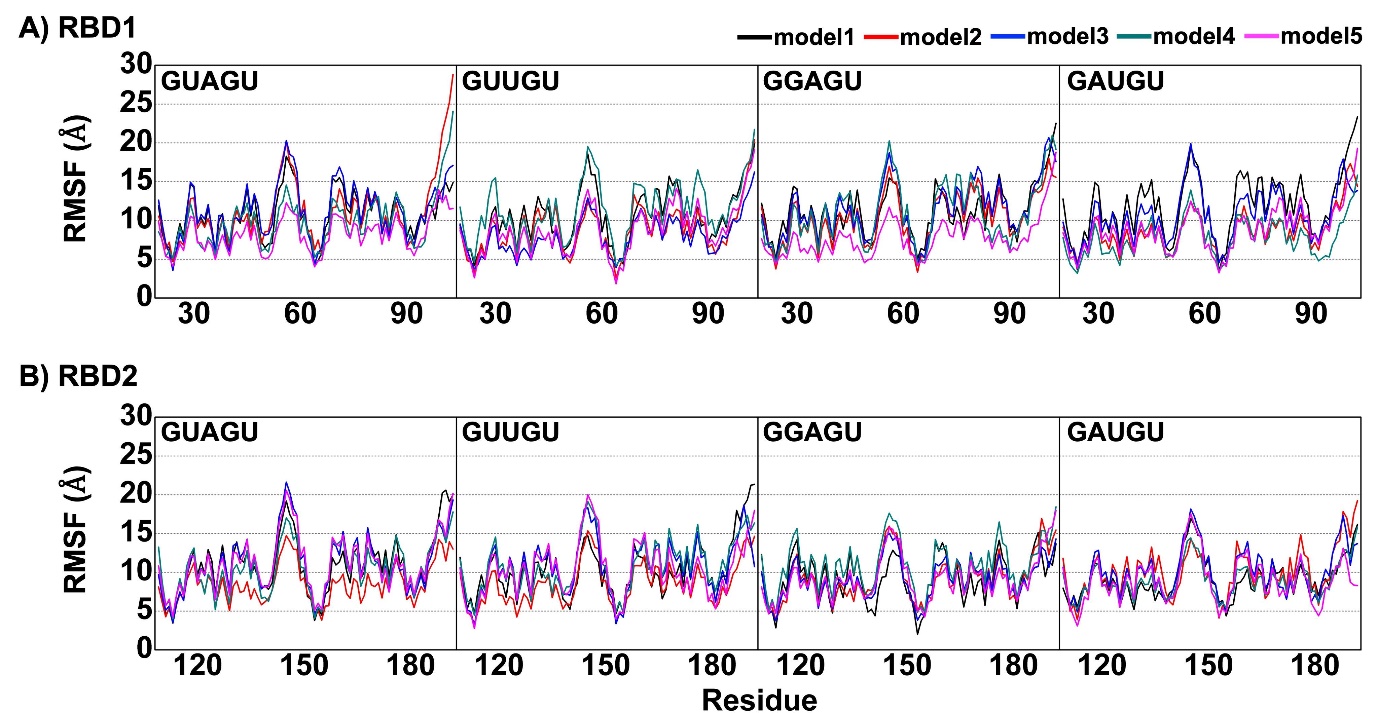


**Figure S7:** RMSF plots of an average MD structure for (**A**) MSI1–RBD1 and (**B**) MSI1–RBD2 in complex with the four RNA pentamers GUAGU, GUUGU, GGAGU, and GAUGU.


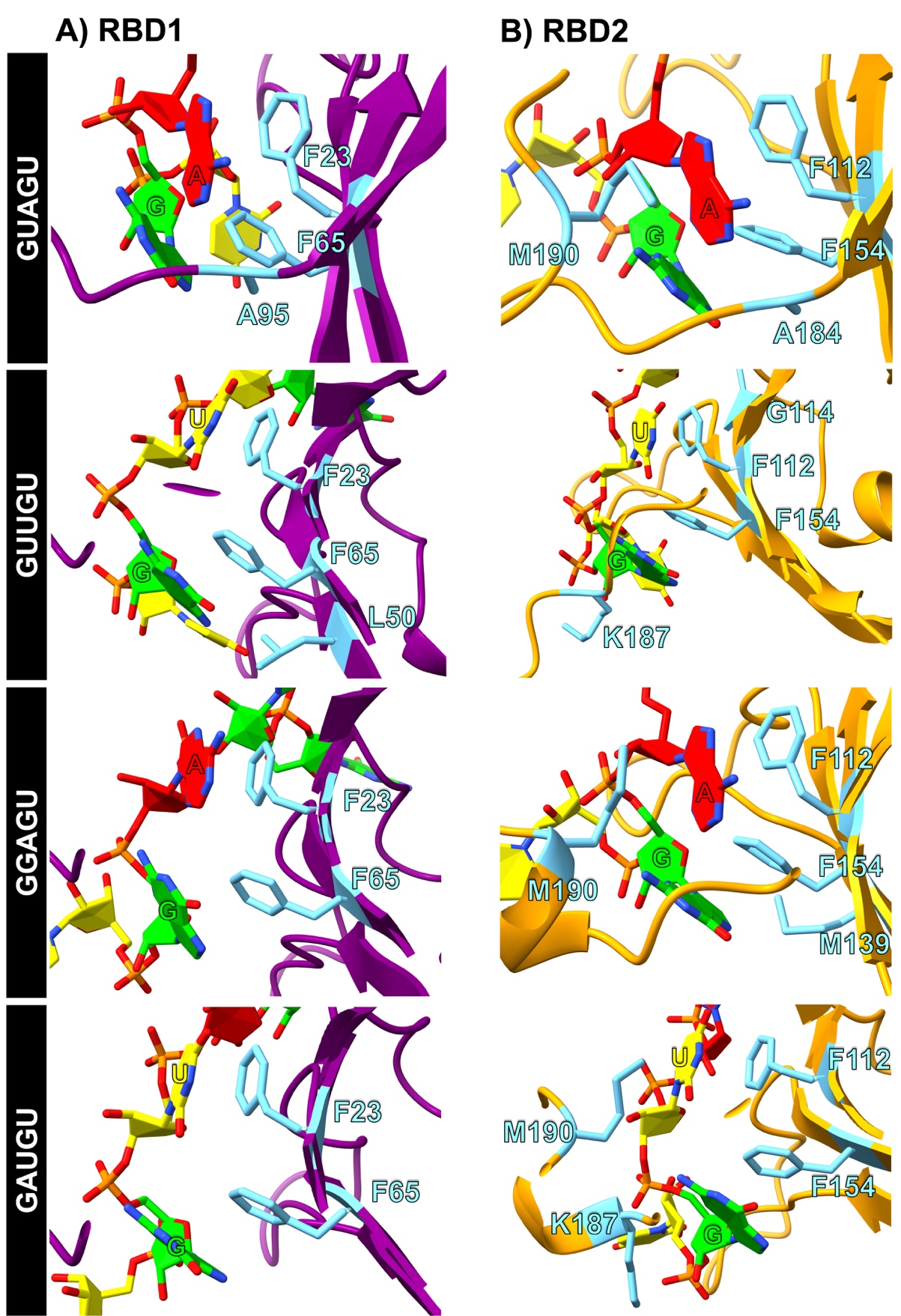


**Figure S8:** Stacking contacts with the studied RNAs at (**A**) MSI1–RBD1 and (**B**) MSI1–RBD2 binding interface. The side chains of binding residues are shown in blue.
